# From Patterns to Pills: How Informatics Is Shaping Medicinal Chemistry

**DOI:** 10.3390/pharmaceutics17050612

**Published:** 2025-05-05

**Authors:** Alexander Trachtenberg, Barak Akabayov

**Affiliations:** Department of Chemistry and Data Science Research Center, Ben-Gurion University of the Negev, Beer-Sheva 8410501, Israel; trachtea@post.bgu.ac.il

**Keywords:** informacophore, drug discovery, inverse cheminformatics, machine learning, data science, medicinal chemistry

## Abstract

In today’s information-driven era, machine learning is revolutionizing medicinal chemistry, offering a paradigm shift from traditional, intuition-based, and often bias-prone methods to the prediction of chemical properties without prior knowledge of the basic principles governing drug function. This perspective highlights the growing importance of informatics in shaping the field of medicinal chemistry, particularly through the concept of the “*informacophore*”. The informacophore refers to the minimal chemical structure, combined with computed molecular descriptors, fingerprints, and machine-learned representations of its structure, that are essential for a molecule to exhibit biological activity. Similar to a skeleton key unlocking multiple locks, the informacophore points to the molecular features that trigger biological responses. By identifying and optimizing informacophores through in-depth analysis of ultra-large datasets of potential lead compounds and automating standard parts in the development process, there will be a significant reduction in biased intuitive decisions, which may lead to systemic errors and a parallel acceleration of drug discovery processes.

## 1. Approaches to Drug Discovery and Development

### 1.1. Classical Drug Discovery

Classical drug discovery follows a structured pipeline of complex and time-consuming steps [1]. The process begins with identifying a biological target, such as DNA, RNA, or a specific protein that plays a particular role in disease development. Thereafter, hit compounds are identified by screening molecules that can interact with the chosen biological target. After the identification of these hit compounds, their chemical structures and drug properties must be optimized to develop lead compounds. Completion of the hit-to-lead process is followed by the preclinical phase, during which the ADMET properties (absorption, distribution, metabolism, excretion, and toxicity), safety, and dosage of promising drug candidates are further evaluated both in vitro and in vivo. For successful candidates, the long process of clinical trials is then begun to evaluate drug safety and effectiveness in humans.

**Figure 1:**
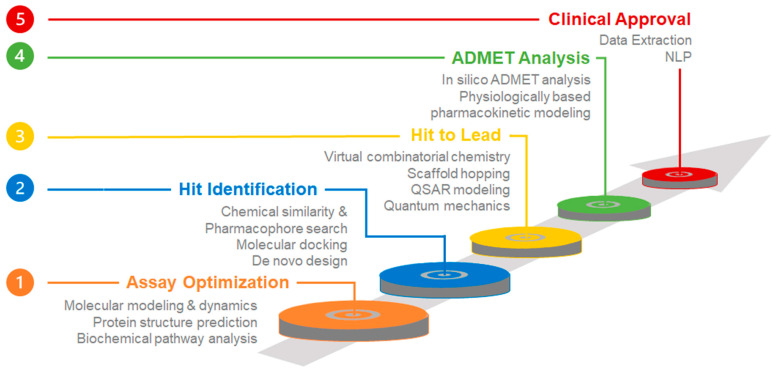
Overview of different steps in the drug discovery pipeline and the corresponding computational tools that may be applied to expedite each step. ADMET—absorption, distribution, metabolism, excretion, and toxicity; QSAR—quantitative structure–activity relationships; NLP—natural language processing.

It has been estimated that the average cost of a classical drug discovery pipeline is USD 2.6 billion and that a complete traditional workflow can take over 12 years [2]. Thus, to shorten discovery timelines, reduce development costs, and improve the odds of clinical success, computational- and artificial intelligence (AI)-based methods have been used to counter the high costs and lengthy timelines that constitute significant bottlenecks in drug development [3]. Figure 1 illustrates the classical drug discovery paradigm, highlighting five key steps in the pipeline: assay optimization, hit identification, hit-to-lead progression, ADMET analysis, and clinical approval. Each of these steps, in turn, narrows the chemical search space for potential drug candidates, as indicated by the varying sizes of the circles in the figure, where the larger the circle, the greater the chemical space. Each step includes an appropriate computer-aided drug design (CADD) method or combination of methods, which may incorporate informatics, modeling, simulation, and/or AI. For instance, the hit identification phase can be dramatically accelerated by implementing CADD methods, such as de novo design, molecular docking, pharmacophore modeling, and chemical similarity searches, to significantly enhance virtual screening or chemical data analysis processes [4].

### 1.2. The Role of Biological Functional Assays in Modern Drug Discovery

While computational tools and AI have revolutionized early-stage drug discovery by enabling rapid identification of potential drug candidates, these in silico approaches are only the starting point of a much broader experimental validation pipeline. Theoretical predictions—such as target binding affinities, selectivity, and potential off-target effects—must be rigorously confirmed through biological functional assays like enzyme inhibition, cell viability, reporter gene expression, or pathway-specific readouts to establish real-world pharmacological relevance [5]. These assays, conducted in vitro or in vivo, offer quantitative, empirical insights into compound behavior within biological systems. They also provide critical data on compound activity, potency, and mechanism of action, thereby acting as an indispensable bridge between computational hypotheses and therapeutic reality. These critical data and insights validate or challenge AI-generated predictions and provide feedback into structure–activity relationship (SAR) studies, guiding medicinal chemists to design analogues with improved efficacy, selectivity, and safety [6]. This iterative feedback loop—spanning prediction, validation, and optimization—is central to the modern drug discovery process [7].

Advances in assay technologies have further strengthened this feedback mechanism. High-content screening, phenotypic assays, and organoid or 3D culture systems offer more physiologically relevant models that enhance translational relevance and better predict clinical success [8]. In this context, biological functional assays are not just confirmatory tools but strategic enablers that shape the direction of both computational exploration and chemical design. This synergy is exemplified in several notable drug discovery case studies:

- Baricitinib, a repurposed JAK inhibitor identified by BenevolentAI’s machine learning (ML) algortihm as a candidate for COVID-19, required extensive in vitro and clinical validation to confirm its antiviral and anti-inflammatory effects, ultimately supporting its emergency use authorization [9].
- Halicin, a novel antibiotic discovered using a neural network trained on a dataset of molecules with known antibacterial properties, enabling the model to identify compounds with potential activity against Escherichia coli. Although the compound’s antibacterial potential was flagged computationally, biological assays were crucial to confirming its broad-spectrum efficacy, including activity against multidrug-resistant pathogens in both in vitro and in vivo models [10].
- Capmatinib, initially developed as an oncology drug, was identified through systems biology and AI as a candidate for antiviral therapy. Its ability to disrupt coronavirus replication was validated through functional assays, highlighting its potential for drug repurposing [11,12].
- Vemurafenib, a BRAF inhibitor for melanoma, was initially identified via high-throughput in silico screening targeting the BRAF (V600E)-mutant kinase. Its computational promise was validated through cellular assays measuring ERK phosphorylation and tumor cell proliferation [13], ultimately guiding SAR efforts to enhance potency and reduce off-target effects.

These cases underscore a fundamental principle in modern drug development: without biological functional assays, even the most promising computational leads remain hypothetical. Only through experimental validation is therapeutic potential confirmed, enabling medicinal chemists to make informed decisions in the iterative process of drug optimization. Biological assays thus form the empirical backbone of the discovery continuum, ensuring that AI-driven innovation translates into real-world medical advances.

### 1.3. Rational Drug Design in Scaffold-Centric Medicinal Chemistry

As computational and biological methods converge to identify promising lead compounds, refining these candidates into viable therapeutics is the next crucial step in the discovery pipeline. This refinement increasingly centers on the molecular scaffold—the minimal structure required for bioactivity—underscoring the importance of rational drug design (RDD). Traditionally, the *pharmacophore* has been used to represent the spatial arrangement of chemical features essential for molecular recognition by a biological target. While the pharmacophore is rooted in human-defined heuristics and chemical intuition, the *informacophore* extends this idea by incorporating data-driven insights derived not only from SARs, but also from computed molecular descriptors, fingerprints, and machine-learned representations of chemical structure. This fusion of structural chemistry with informatics enables a more systematic and bias-resistant strategy for scaffold modification and optimization.

Feeding the essential molecular features of the informacophore into complex ML models can offer greater predictive power, but also raises challenges of model interpretability. Unlike traditional pharmacophore models, which rely on human expertise, machine-learned informacophores can be challenging to interpret directly, with learned features often becoming opaque or harder to link back to specific chemical properties. Despite these challenges, hybrid methods—guided by medicinal chemists—are emerging to combine interpretable chemical descriptors with learned features from ML models [14,15]. helping to bridge this interpretability gap. By grounding machine-learned insights in chemical intuition and data-driven patterns, informacophores can better understand how specific chemical features influence biological activity. Thus, they offer the potential to become a key element of modern RDD strategies, offering a more efficient and scalable path from discovery to commercialization than traditional intuition-led approaches [16].

RDD was first formalized in the 1950s, when it became possible for theoretical insights into drug-receptor interactions and hands-on drug testing to continuously reinforce one another, making RDD achievable [17]. In the 1980s, RDD acquired the status of the methodological ideal, following the successful development—in the 1970s—of drugs such as lovastatin (cholesterol lowering) and captopril (antihypertensive), which remain in clinical use until the present [18]. In RDD, molecular modeling is used in conjunction with optimization cycles that rely on considerations of SARs to strategically modify functional chemical groups with the aim of improving the effectiveness of a drug candidate [19].

Curiously, this well-established method has its roots in the pioneering work of Langmuir over a century ago [20], in which the functional groups of a scaffold molecule were altered, but the essential physicochemical properties of the molecule were maintained. In medicinal chemistry, the basic principles guiding the design of such molecules—known as bioisosteres—have not changed much since Langmuir’s time [21]. The process of bioisosteric replacement involves finding the balance between maintaining the desired biological activity of a molecule and optimizing the drug-related properties that influence its efficacy, such as solubility, lipophilicity, stability, selectivity, non-toxicity, and absorption. In practice, bioisosteric replacement involves the use of limited and sometimes unstructured data and, as such, often relies on the intuition of a highly experienced chemist to simplify the decision-making path, say, as to the preferable sites for efficient chemical modifications on the scaffold molecule. Such intuition stems from the chemist’s experience in visual chemical-structural motif recognition, and its association with retrosynthetic routes and pharmacological properties. Therefore, medicinal chemists are required to address challenges related to pattern recognition in their decision-making path toward the ultimate structure of a drug molecule.

### 1.4. Medicinal Chemistry in the Big Data Era

Humans have a limited capacity to process information, which forces them to use heuristics [22]. In contrast, ML algorithms that depend on extensive data repositories can efficiently process vast amounts of information rapidly and accurately. This ability is beyond the capacity of any individual, no matter how expert s/he may be, to find hidden patterns. In this respect, medicinal chemists can benefit from computer-guided data analysis to inform objective and precise decisions, enabling the prediction of biologically active molecules.

The development of ultra-large, “make-on-demand” or “tangible” virtual libraries has significantly expanded the range of accessible drug candidate molecules. These libraries consist of compounds that have not actually been synthesized but can be readily produced. For example, the chemical suppliers Enamine [23] and OTAVA [24] offer 65 and 55 billion novel make-on-demand molecules, respectively.

To screen such vast chemical spaces, ultra-large-scale virtual screening for hit identification becomes essential, since direct empirical screening of billions of molecules is not feasible. Hert et al. [25] showed that for high-throughput screening of a library of the order of 10^6^ molecules to succeed in returning active molecules, the library’s molecules would have to be biased towards “bio-like” molecules, namely, biologically relevant compounds (such as metabolites, natural products, and drugs that mimic these substances) that proteins have evolved to recognize [26].

Due to the vast chemical space represented by tangible libraries, their bias toward bio-like molecules is much lower than that in “in-stock” libraries, as demonstrated in a study by Lyu et al. [26]. Furthermore, that study showed that as the size of the tangible library increased, the number of better-fitting molecules also increased, with docking scores improving in a log-linear fashion with library size. Additionally, the researchers studied five ultra-large-library docking campaigns and found out that thousands of high-ranking molecules, including experimentally active compounds, such as inhibitors, were notably dissimilar to bio-like molecules.

In summary, the researchers obtained high-ranking unique drug candidate molecules, at the cost of vastly exhaustive and expensive computational docking testing. Nonetheless, it is important to stress that in this case, medicinal chemists would not even have suggested those high-ranking molecules as candidates for hit identification, since their dissimilarity to bio-like structures would have contradicted the heuristics commonly applied in their work.

While ML models offer unprecedented capacity to analyze large datasets and explore expansive chemical spaces, they are not immune to bias. In fact, when trained on historical bioactivity data, which is itself shaped by human preferences and experimental feasibility, ML algorithms can inadvertently reinforce existing biases toward well-explored, bio-like scaffolds. This can lead to the underrepresentation of structurally novel yet pharmacologically promising areas of chemical space. Moreover, many ML-driven drug discovery pipelines rely on molecular descriptors or fingerprints that are themselves biased by past datasets, creating feedback loops that marginalize atypical but viable chemical motifs. Thus, despite the theoretical reach of ultra-large virtual libraries, the effectiveness of ML in venturing into less-charted chemical regions depends heavily on the diversity and representativeness of the training data and deliberate efforts to avoid overfitting to “drug-like” norms.

## 2. Improving the Decision-Making Process in Drug Development

### 2.1. The Ability to Predict

The behavioral economists Kahneman and Tversky demonstrated how humans use two paths to solve problems: One is fast and intuitive, while the other is slow and requires analytical thinking [27]. In drug development, both these paths can be considerably improved by predictive models trained on datasets of molecules labeled with activity scores, providing more accurate decisions in the first path and speeding up the decision-making process in the second. To illustrate these ideas, which provide support for informed decision-making in the design of therapeutics tailored to specific biological targets, let us examine three examples. The first one is a compelling example of ML’s predictive power, where Insilico Medicine used generative deep learning models to design novel inhibitors of the SARS-CoV-2 main protease (Mpro). These AI-generated lead compounds were not only chemically feasible but also synthesized and experimentally validated, demonstrating nanomolar inhibitory activity and favorable pharmacological properties [28]. This end-to-end application—from target selection through molecule generation to experimental validation—highlights how predictive models can accelerate the discovery of viable therapeutic candidates tailored to complex biological targets, significantly reducing development timelines.

The second example centers on quantitative structure–activity relationships (QSARs) analysis, a computer-aided method used in the hit-to-lead process. This methodology (based on the seminal publication of Hansch et al. that appeared in 1962 [29]) is utilized to predict differences in the biological activities of chemical compounds by correlating them with a quantitative description of variations in their structures [30]. By leveraging deep learning methods, which have been employed to develop advanced QSAR models (designated as “deep QSAR models”), quantum mechanics calculations can now be performed more efficiently, reducing computational time and improving the accuracy of the relevant QSAR models [31]. This enhancement, in turn, contributes to higher accuracy in calculating ligand binding affinity and molecular properties and ultimately improves the ability to identify promising drug candidates [31]. For example, Rufa et al. [32] used a hybrid approach combining ML with molecular mechanics to achieve a root mean square error (RMSE) of 0.47 kcal/mol for binding free energy predictions—nearly halving the error (originally 0.97 kcal/mol) of conventional molecular mechanics alone. Deep QSAR models have recently been combined with chemical language models, either as separate external tools to rank the generated molecules by activity or as model-intrinsic scoring functions to guide chemical structure generation towards molecules with particular properties [33,34,35].

We take the third example from the ADMET analysis step of drug discovery, where physicochemical properties critical to pharmacokinetics and formulation are optimized. One such property is the octanol−water partition coefficient (log P)—a key measure of lipophilicity. It indicates how a compound distributes between aqueous and lipid environments, thereby influencing its behavior in biological systems. A drug with a high log *p* value typically exhibits enhanced membrane permeability due to favorable partitioning into lipid bilayers, but this may also lead to reduced aqueous solubility and suboptimal bioavailability. Conversely, compounds with low log *p* values may struggle to cross lipid membranes, limiting absorption. The log *p* value of a compound also correlates with other drug-relevant properties, such as excretion, metabolic stability, and tissue distribution [36]. Since it may be difficult to determine log P experimentally for particular compounds and for some ranges of log P, quantum-mechanics-based tools have emerged as a preferred alternative to existing empirical models [37]. However, they demand a substantial computational cost of around 1 h per compound [37]. To address this problem, Lewis et al. [37] trained a message-passing neural network model, known as Chemprop, on both a public dataset of compounds and a Novartis in-house dataset, to obtain a computationally affordable quantum-mechanics-based predictive model of drug lipophilicity values for hundreds of compounds per second. Their Chemprop-based model achieved mean absolute errors (MAEs) of 0.44 and 0.34 log units on scaffold-split test sets of public and in-house datasets, outperforming traditional regression models and demonstrating scalability and predictive robustness. In addition, using learning curves, they showed that additional training data for both the public and in-house datasets could probably further decrease the test set error. It would thus seem that their model could be used to pre-screen large libraries of compounds, making it useful in the decision-making process of prioritization of candidate compounds for full quantum mechanics calculations of the likelihood that a particular compound would succeed in clinical studies.

### 2.2. Man vs. Machine

**Figure 2:**
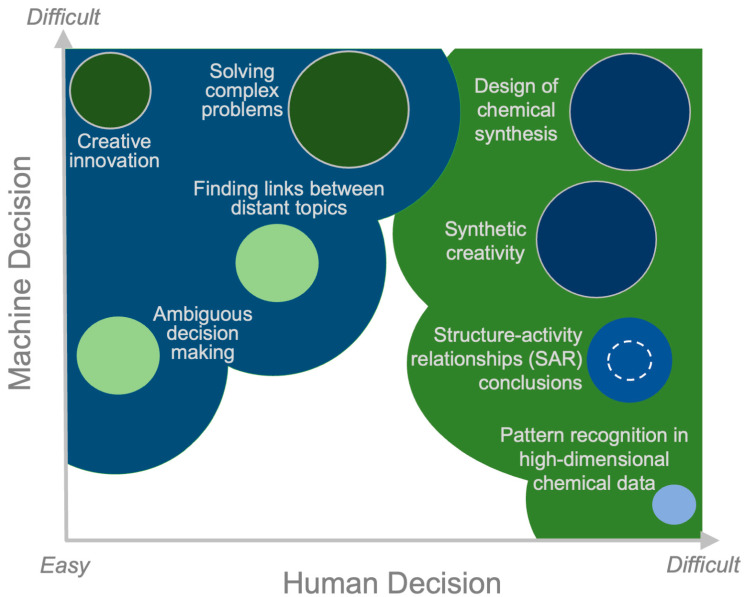
The complexity of drug design and medicinal chemistry tasks (depicted by circles) for machine learning (ML) algorithms vs. human capabilities. The meanings of the circle sizes are as follows: Larger circles represent more complex ML algorithms or require higher human expertise. Smaller circles represent simpler ML algorithms or require less human expertise. The meanings of the circle shade colors are as follows: Blue represents the size of the dataset needed for training ML algorithms. Darker blue means that larger datasets are required for better decision-making. Lighter blue means that smaller datasets can be used. Green represents the level of human expertise needed. Darker green means that higher expertise is required to accomplish the task. Lighter green means that lower expertise or a more novice level will accomplish the task.

Humans have the creative ability to develop new hypotheses and ideas that are not based on prior information or observations [38]. In the human decision-making process, this ability is enhanced by integrating considerations beyond the capacity of algorithms, for example, ethical rules, non-specific drug effects, and even long-term strategies in drug development. Figure 2 presents a graphical comparison of this human capability with that of ML in terms of the relative difficulty of performing different tasks related to drug design and medicinal chemistry. Each task—for human vs. computer-aided capabilities—is represented by a circle, whose size indicates the complexity of the calculation involved vs. the complexity of the algorithm (the larger the circle, the more complex the task) and whose shade of color represents the level of expertise required vs. the size of dataset needed for the ML algorithm to perform effectively. For instance, the shade of the blue circle representing the “conclusions drawn from SARs” indicates that medium-sized datasets can be utilized (intermediate shade of blue) and that the conclusions can be drawn by simple linear ML algorithm (enclosed within the dashed circle) or more complex ones (non-linear algorithms to neural networks) [39]. In contrast, the large dark blue circles for the “design of chemical synthesis” or “synthetic creativity”, i.e., generating new molecules, require complex ML algorithms and large datasets [40,41].

As is apparent from the above discussion, humans have a clear advantage in solving complex problems that require a holistic overview of systems or adaptations to changing, unpredictable, or unfamiliar situations, such as changes that must be made to comply with updated regulations for particular drugs. In contrast, the advantage of ML algorithms lies in the ease with which they can identify patterns in high-dimensional chemical data, even if the patterns are weak and hidden in a large dataset [42]. The medicinal chemist can, therefore, leverage ML as a complementary tool to effectively utilize big data to reveal patterns and hidden features and, hence, to establish rules to build generalized, fast, and accurate models. It should, nonetheless, be remembered that while ML provides valuable insights and information, it cannot completely replace the expertise, creativity, and intuition of the medicinal chemist. Even validated ML models often overlook the specific context of the drug discovery project and do not take into account the downstream assays that are crucial for experimentally validating the model’s predictions. For example, if a model predicts toxicity, it is essential to understand what this prediction means in terms of the type of disease (e.g., lifestyle-related illness or terminal cancer), dosage, target tissue, etc. In this context, the experience and intuition of a medicinal chemist will play a crucial part in assessing a model’s predictions. These ideas are reflected in Table 1, which summarizes the capabilities and limitations of ML and human expertise in drug discovery tasks.

Furthermore, as AI systems play an increasing role in molecule design, their integration into the drug development pipeline must be accompanied by a careful examination of both ethical and regulatory frameworks. Current regulatory bodies, such as the FDA and EMA, are beginning to adapt their guidelines to assess AI-generated outputs, but there remains uncertainty regarding the validation, transparency, and accountability of models that operate as “black boxes” [43]. Without explainability, it becomes difficult to justify how and why certain molecules were selected for further development, posing a challenge to regulatory approval. Ethically, there are concerns about delegating creative and high-stakes decisions to machines, especially in areas where unintended consequences, such as off-target effects or biases in training data, could have significant impacts on patient safety [44]. Additionally, the rapid pace of AI innovation can outstrip the ability of governance structures to evaluate risks and ensure equity, such as fair access to AI-accelerated therapies. This highlights the need for explainable AI (XAI) mechanisms, which help improve transparency and ensure that AI decisions are understood, reducing risks related to algorithmic biases and increasing trust in AI systems used in drug discovery [43,44]. Ultimately, while AI can serve as a powerful complement to human expertise, it also necessitates a collaborative approach in which the human expert remains the final arbiter of judgment, balancing machine-generated suggestions with ethical responsibility, contextual knowledge, and regulatory foresight.

## 3. Machine Decision Making

### 3.1. Chemical and Visual Descriptors

As mentioned above, ML algorithms can easily identify patterns in molecular structures that would improve the function of particular drug molecules [42]. An essential aspect of analyzing these patterns begins with a molecular representation of the chemical data [45]. One of the “tools” that has been applied for this purpose is graph theory, but the representation of molecules by graphs composed of vertices (atoms) and connecting edges (bonds) is sparse due to the limited connectivity between the atoms in the molecules. Additional drawbacks of graph-based representations are that they lack uniqueness (since different connectivity tables can represent the same chemical structure) and they are not adequate for representing certain types of molecules, particularly aromatic and organometallic compounds [46].

In seeking a more satisfactory means for molecular representation of chemical data, Akabayov and his colleagues recently explored various feature extraction methods to train supervised models that predict the binding of small molecules to RNA targets [47,48]. To extract chemical descriptors and geometrical patterns that aid in the understanding—and prediction—of molecule–RNA binding affinities, those studies analyzed various molecular representations (including SMILES strings—simplified molecular input line entry system and pictorial representations) by employing Lasso regression, decision tree classifiers, and convolutional neural network (CNN) models. While CNNs applied to molecular images can uncover complex spatial and visual features, their outputs are often considered less chemically interpretable compared to graph-based or descriptor-driven methods [49]. To address this, the mentioned study [47] has integrated CNN-derived visual features with chemically defined descriptors, such as solubility, molecular regularity, and counts of hydroxy and amino groups, thereby anchoring model predictions in chemically meaningful patterns. Since then, emerging model interpretability techniques, such as saliency mapping and feature attribution methods, can help identify which image regions drive predictions [50,51], improving the chemical relevance of CNN-based insights. These integrative approaches exemplify the evolving notion of the informacophore, which extends traditional pharmacophore modeling by combining human-interpretable chemical features with data-driven, ML representations to enhance understanding of molecular recognition across diverse biological targets. Collectively, these studies facilitate a unique understanding of the interplay between molecular structure and biological activity, and, in the case of small molecules binding to RNA targets, the identification of intrinsic properties of potent inhibitors.

### 3.2. Reducing the Complexity of a Molecule

Efficient exploration of the vast, novel chemical landscapes enhances the prospects for early-stage discovery of effective drug candidates [52]. However, the chemical search space for pharmacologically relevant small molecules is enormous [53]—being of the order of 10^60^. This sheer size makes it extremely challenging to efficiently identify candidate compounds with the desired bioactivity from the full search space. This inefficiency is due to the very limited chemical space covered by small molecule libraries [54,55], which results in a low hit rate in high-throughput screening (HTS) of libraries of the order multimillions of compounds.

A powerful approach to aid in easing this problem is fragment-based drug discovery (FBDD), which offers several advantages over HTS campaigns [56]. FBDD leverages the concept that small chemical fragments (with low molecular weights of less than 300 Da) that bind weakly to specific sites on a target protein can be elaborated into larger more complex molecules that can serve as potent lead candidates [57]. The premise underlying FBDD is that in HTS studies higher hit rates will be obtained for such small fragment molecules than for full drug-like molecules possessing functional groups that may pose steric hindrance or electrostatic repulsion in a binding site [58,59]. Thus, FBDD facilitates the exploration of a broader chemical space with fewer fragment compounds. However, it is also important to notice that fragments often exhibit very weak binding affinities, making them difficult to detect without sensitive biophysical methods such as X-ray crystallography, NMR spectroscopy, and isothermal titration calorimetry (ITC) [60]. Additionally, many fragment hits face solubility issues, and their synthetic elaboration into drug-like molecules can be non-trivial. These challenges can hinder the optimization process from fragment to lead compound [61].

A practicable strategy to reduce the vast chemical search space for drug-sized (small) molecules with enhanced bio-activity is the use of the same scaffold as that obtained by fragment-based screening [62]. In applying this strategy, the Akabayov group first used NMR transverse relaxation times (T2 relaxation) as a fragment screening method to identify a hit functional chemical group that binds to an RNA target [48,63]. The group then followed a hit-to-lead workflow that included a two-step computational optimization [63] with the aim of increasing the size of the molecule and, at the same time, extending the network of weak interactions between the small molecule and the RNA target. This approach enhanced both the specificity and strength of small molecule–RNA binding, moving the compound closer to a viable lead. By starting from a validated fragment scaffold, it was possible to efficiently navigate the chemical space to design drug-sized molecules with improved bioactivity.

### 3.3. The Informacophore and Inverse Cheminformatics

In computationally aided drug discovery, data-driven algorithms are used to reveal the features critical for the activity of a small molecule in binding to a biological target, such as a protein or receptor [64]. These key features, which comprise the chemical and structural fingerprints of a small molecule, form the informacophore, which represents the minimal chemical structure required to induce biological activity. Conceptually, the informacophore can be viewed as a refined, function-oriented scaffold—a minimal framework that retains only the essential elements for target binding.

The informacophore is often slightly larger than a molecular fragment, yet it contains only those atoms or functional groups necessary for molecular recognition, acting much like a “key” that fits a specific “lock”. For instance, if a compound is found to bind strongly to a particular protein, the informacophore of that compound consists of the minimal set of atoms or functional groups responsible for this interaction. By analyzing a collection of molecules with shared informacophore features, researchers can cluster compounds with similar biological activity profiles and predict the activity of new molecules based on their informacophore structure. This approach dramatically narrows the chemical search space, enabling more efficient virtual screening and compound optimization. Cheminformatics tools, which extract molecular descriptors from chemical structures, play a crucial role in identifying informacophores and predicting bioactivity. By analyzing the informacophore of a molecule, through the extraction of structural descriptors and visual patterns from chemical data, researchers can model and anticipate its biological activity. For example, the informacophore of Imatinib, an established cancer drug, can be computationally derived and used to predict the activity of other compounds that share similar informacophore-derived features and are likely to exhibit comparable binding profiles to the same target.

**Figure 3:**
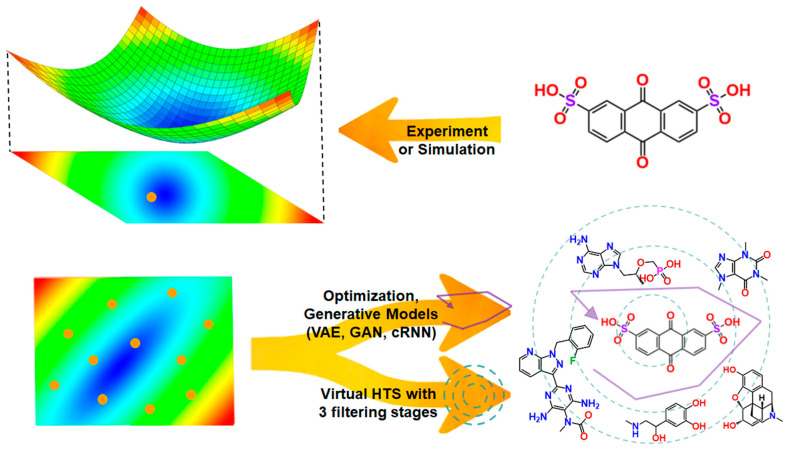
Schematic representation of the different approaches to molecular design. The direct approach (**top**) leads from chemical space to the properties of the molecule, whereas inverse design (**bottom**) proceeds from the desired properties to chemical space. **Left**: a 2D projection of a high-dimensional property landscape illustrates functional space; **right**: example molecular structures. The figure was redrawn based on ref. [65].

Conversely, in the reverse process, known as inverse cheminformatics, novel bioactive molecules may be created by applying design principles based on extracted chemical patterns [65]. This reverse process allows scientists to design new compounds systematically, guided by a detailed understanding of the chemical properties and biological interactions derived from cheminformatics, ultimately leading to the development of effective and targeted therapeutic agents [66]. Figure 3 compares the direct and inverse design paradigms, illustrating the chemical and functional space transition. In the direct design approach, one starts from chemical structures and evaluates their corresponding properties. Conversely, inverse design begins with desired functional properties and searches for chemical structures that meet those criteria.

For inverse design, two main pathways are commonly employed: (i) Virtual screening of functional space to identify optimal combinations of molecular properties—often through large databases or predictive models. For example, in a recent study, Menacer et al. [67] introduced a novel methodology integrating inverse-QSAR with molecular docking for the de novo design of SARS-CoV-2 main protease inhibitors. By utilizing simple, reversible descriptors, their approach addressed the limitations of traditional inverse-QSAR techniques and enabled the generation of novel scaffold-based structures with predicted activity. This approach balances predictive accuracy with interpretability by ensuring that the descriptors used in the inverse-QSAR model retain chemical meaning. (ii) Optimization through the traversal of functional space using generative models, such as variational autoencoders (VAEs), generative adversarial networks (GANs), and conditional recurrent neural networks (cRNNs). Table 2 summarizes the key characteristics of these generative model architectures explored in recent molecular design efforts. Each model type offers unique advantages in how molecules are represented, generated, and optimized.

The complexity of such generative models raises concerns about their lack of direct interpretability [66]. These models may generate highly complex molecular structures, but understanding the underlying decision-making process remains a significant challenge. Researchers are beginning to address this by developing hybrid approaches that combine deep learning with traditional cheminformatics to ensure the generated molecules are not only novel but also chemically plausible [68,69]. However, despite their promise, generative approaches face significant challenges in translating in silico designs into viable drug candidates. A major limitation lies in the failure rate of generated molecules—many may be chemically unstable, synthetically inaccessible, or biologically irrelevant [70]. For instance, molecules sampled from latent spaces can satisfy mathematical constraints but fail to meet key medicinal chemistry criteria, such as Lipinski’s rules, toxicity thresholds, or reactivity filters. Furthermore, synthetic feasibility is a critical bottleneck: a molecule’s theoretical desirability is insufficient unless it can be practically synthesized [71]. Recent efforts have introduced retrosynthesis-aware filters and synthetic accessibility scoring (SAS) functions into generative workflows, but these tools remain imperfect [72].

Additional post-generation filtering is often required to assess drug-likeness and eliminate structures that, while novel, may fall outside the boundaries of known pharmacophores. For instance, models such as MolFilterGAN have shown promise in triaging AI-designed molecules by integrating synthetic feasibility and biological relevance [73]. Furthermore, retrosynthesis-aware scoring tools like RAscore have made it possible to incorporate synthesizability directly into generative processes, helping bridge the gap between theoretical generation and practical chemistry [72]. These developments underscore the necessity for inverse design pipelines to integrate comprehensive evaluation steps that prioritize candidates not only for novelty and predicted activity, but also for synthesizability, pharmacological relevance, and safety.

An emerging and highly complementary strategy to enhance the performance of these generative models—especially in scenarios with limited labeled data—is transfer learning. Transfer learning enables models trained on large, general-purpose chemical datasets to be fine-tuned for more specific, data-scarce applications such as RNA-targeted ligand prediction. This approach has shown that even complex property prediction tasks can benefit from knowledge learned in adjacent domains, especially when leveraging shared latent representations. For instance, recent studies have demonstrated that transfer learning using latent variables, such as those derived from autoencoders, can significantly improve property prediction in low-data regimes without sacrificing performance in high-data settings [74]. Importantly, this strategy is not limited to VAEs. GANs can incorporate transfer learning by initializing the generator and discriminator with weights pretrained on large molecular datasets, enhancing their ability to generate chemically valid structures from limited task-specific data. Similarly, cRNNs can be pretrained on large corpora of SMILES strings and later conditioned on task-relevant features for fine-tuned generation of molecules that meet specific property constraints. By embedding transfer learning within these generative frameworks, researchers can build models that not only generalize better across diverse chemical tasks but also accelerate discovery in niche domains where experimental data is inherently limited [74].

**Table 2:** Overview of generative model architectures commonly used in molecular design. Each model type is characterized by its core purpose in handling chemical representations, a defining feature that underlies its generative capability, and representative studies that have contributed to its development and application in drug discovery or molecular optimization.

Model Type	Chemical Purpose	Key Feature	Referenced Studies
VAE (Variational Autoencoder)	Encode molecules into a continuous latent space and decode to generate novel chemically meaningful structures	Smooth latent space allows interpolation and optimization of molecular properties	Gómez-Bombarelli et al. [75]
GAN (Generative Adversarial Network)	Learn to generate valid molecular structures by training a generator against a discriminator	Adversarial training enables generation of novel molecules with predefined bioactivity profiles	Zhavoronkov et al. * [28] Kadurin et al. [76]
cRNN (Conditional Recurrent Neural Network)	Learn physicochemical or structural characteristics to generate molecules conditioned on desired properties	Sequential generation of molecules guided by learned property constraints	Kotsias et al. [77] Mohapatra et al. [78]
Flow-based Neural Network	Model the exact likelihood of molecular data for reversible generation	Enables bidirectional mapping between molecule and latent space with tractable likelihoods	Hu Wei [79]

* The study by Insilico Medicine’s, discussed earlier in Section 2.1, is marked with an asterisk.

## 4. Outlook

The application of ML and cheminformatics in drug design is advancing rapidly, due both to significant improvements in data collection, storage capacity, and computational power and to the availability of specialized ML algorithms. This progress has enabled the identification of key molecular features that enhance the accuracy of predictive models. The current shift towards more efficient and informed drug design processes may thus be regarded as the synergetic combination of computational models and the expertise of medicinal chemists.

However, as AI-driven methods become more integrated into drug discovery, it is essential to acknowledge their limitations. These include challenges such as model explainability [80], data bias, and the risk of underfitting or overfitting [81]. AI models are only as good as the data they are trained on, and biases in data can lead to inaccurate or skewed predictions [82]. Additionally, the lack of transparency in some ML models can hinder the ability to interpret how decisions are made, raising concerns about trust and accountability in drug development [80]. Therefore, despite their immense potential, AI and ML tools must be used with caution and always in conjunction with human oversight. Ongoing validation—especially through laboratory experiments—and careful monitoring are crucial to ensure that these technologies lead to meaningful and safe therapeutic advancements. As computational technologies continue to evolve, their effective integration with expert human judgment will be essential, creating a synergistic dynamic that not only accelerates drug discovery but also enhances the reliability and precision of therapeutic interventions.

To conclude, AI and ML are not here to replace human ingenuity in medicinal chemistry, but to augment it. The future of drug discovery lies in a collaborative approach, where computational models and human expertise work together to push the boundaries of what is possible. Advanced AI techniques will enhance the capabilities of medicinal chemists, allowing them to make more informed, precise, and innovative decisions. The path forward is one where AI and human insight converge to revolutionize the field, making drug discovery faster, more accurate, and ultimately more impactful. Looking ahead, we propose two possible key evolution routes that will drive the future of drug discovery:

- The first involves FBDD and make-on-demand compound libraries, which will enable virtual searches for bioactive compounds rather than relying on traditional HTS. This shift will streamline the drug discovery process, reducing the need for extensive physical screening, as was the case in the past 25 years [83].
- The second route focuses on advancements in molecular representations. As traditional representations like SMILES and graph-based representations fail to capture the complexity of large molecules [84], there is a need for more sophisticated representations that improve the accuracy of property predictions and virtual screening. These developments will enhance the performance of ML algorithms and help produce more reliable and precise drug discovery outcomes.

Together these routes will work in tandem to improve the power and reliability of ML algorithms in drug discovery. Furthermore, an understanding of how molecular representations influence model interpretability [85] could provide valuable insights for medicinal chemists, allowing them to leverage AI-driven decisions to inform their own expertise and judgment. As the integration of AI into drug discovery continues to evolve, these advancements will ensure that human oversight remains central, with AI serving as a powerful tool for enhancing, rather than replacing, human intuition and expertise.

## Figures and Tables

**Table 1:** Comparison of ML vs. human expertise in drug discovery tasks. This table highlights the strengths and limitations of both ML models and human expertise across key tasks in drug discovery, including molecule design, SAR analysis, toxicity prediction, chemical synthesis planning, biological target identification, and clinical trial design.

Task	ML Capabilities	ML Limitations	Human Expertise Capabilities	Human Expertise Limitations
Molecule Design/Synthetic Creativity	Rapidly generates novel molecular structures from extensive datasets. Efficient in exploring vast chemical spaces. Identifies subtle, non-obvious molecular features.	Struggles with understanding context-specific details, such as disease nuances. Lacks insight into biological or off-target effects. May not propose innovative designs beyond existing data.	Can generate truly novel ideas and hypotheses. Integrates biological, therapeutic, and regulatory contexts. Has intuition for unexpected solutions.	Time-consuming. Limited by experience in specific areas. Limited ability of using large datasets for pattern recognition.
SAR analysis	Analyzes high-dimensional datasets to identify pattern. Quickly generates predictive models to assess activity across compounds.	Models can fail with noisy data. The models may struggle with complex relationships between molecular features. Performance relies on training data quality.	Expertise in interpreting contextual nuances of SAR data. Flexible in assessing novel relationships or unexpected biological effects.	Limited ability to analyze large datasets manually. Biases may influence interpretation.
Toxicity Prediction	Capable of analyzing extensive datasets of toxicity reports to forecast potential toxicological effects.	Missing rare or context-specific toxicity risks. Lacking biological insight in certain cases. Being unable to consider contextual factors such as patient-specific variables like age and comorbidities.	Contextual understanding of toxicity, such as tissue type and dosage. Ability to interpret complex, multifactorial interactions that lead to toxicity.	Insights may be generated slowly, slowing down decision-making. May lead to missing emerging trends or new patterns and hinder innovation and adaptability.
Chemical Synthesis Planning	Can predict viable synthetic routes using comprehensive datasets from prior reactions. Identifies efficient reaction conditions and reagents.	Being constrained by existing data, which may prevent the generation of truly novel synthesis pathways. Struggles with complex, multi-step synthesis that requires intuition or innovative thinking.	The ability to improvise when synthetic routes are ineffective. An intuitive understanding of how to adapt synthesis methods based on specific compound structures.	Struggles with scalability for a large number of compounds. Resource constraints in synthetic efforts can limit creativity.
Biological Target Identification	Can analyze extensive genomic and proteomic datasets to identify potential drug targets. Employs a data-driven and systematic approach to searching for candidate targets.	Lack of contextual understanding of disease biology and patient variability. May overlook rare or novel biological targets that are not represented in the training data.	Deep understanding of biological mechanisms in specific diseases. Ability to contextualize target discovery through biological insight and experience.	Often struggle with large-scale data integration. Slower to recognize new or unexpected targets.
Clinical Trial Design	Can optimize trial designs by analyzing data of a certain statistical distribution and identifying factors influencing patient outcomes.	Challenge in integrating individual patient differences or ethical considerations. Lacks the ability to generate human-centered solutions based on societal needs.	Ability to design trials with a focus on human factors, ethics, and patient variability. Adapts trial design to emerging data and real-world conditions.	Time-consuming and resource-intensive. Can be inflexible when adapting existing designs to new conditions.
